# Harnessing Metal‐Organic Frameworks for NIR‐II Light‐Driven Multiphoton Photocatalytic Water Splitting in Hydrogen Therapy

**DOI:** 10.1002/advs.202405643

**Published:** 2024-08-09

**Authors:** Xin Lu, Xinlei Yu, Bo Li, Xianshun Sun, Longjiu Cheng, YuanZhong Kai, Hongping Zhou, Yupeng Tian, Dandan Li

**Affiliations:** ^1^ Institutes of Physical Science and Information Technology Faculty of Materials Science and Engineering School of Chemistry and Chemical Engineering School of Life Sciences Key Laboratory of Structure and Functional Regulation of Hybrid Materials Ministry of Education Anhui University Hefei 230601 P. R. China

**Keywords:** hydrogen therapy, metal‐organic framework, multiphoton absorption, near‐infrared light, photocatalytic

## Abstract

The construction of near‐infrared (NIR) light‐activated hydrogen‐producing materials that enable the controlled generation and high‐concentration release of hydrogen molecules in deep tumor tissues and enhance the effects of hydrogen therapy holds significant scientific importance. To address the key technical challenge of low‐efficiency oxidation–reduction reactions for narrow‐bandgap photocatalytic materials, this work proposes an innovative approach for the controllable fabrication of multiphoton photocatalytic materials to overcome the limitations imposed by traditional near‐infrared photocatalysts with “narrow‐bandgap” constraints. Herein, an NIR‐responsive multiphoton photocatalyst, **ZrTc‐Co**, is developed by utilizing a post‐synthetic coordination modification strategy to introduce hydrogenation active site Co^II^ into a multiphoton responsive MOF (**ZrTc**). The results reveal that with the introduction of the Co^II^ site, electron–hole recombination can be efficiently suppressed, thus promoting the efficiency of hydrogen evolution reaction. In addition, the integration of Co^II^ can effectively enhance charge transfer and improve static hyperpolarizability, which endows **ZrTc‐Co** with excellent multiphoton absorption. Moreover, hyaluronic acid modification endows **ZrTc‐Co** with cancer cell‐specific targeting characteristics, laying the foundation for tumor‐specific elimination. Collectively, the proposed findings present a strategy for constructing NIR‐II light‐mediated hydrogen therapeutic agents for deep tumor elimination.

## Graphical abstract

This work presents a multiphoton responsive metal‐organic framework for near‐infrared‐II (NIR‐II) light‐driven photocatalytic hydrogen (H_2_) generation using nicotinamide‐adenine dinucleotide (NADH) as a sacrificial agent for tumor elimination. This approach provides a new design for multiphoton photocatalysts and offers a groundbreaking approach that can overcome the limitations imposed by traditional near‐infrared photocatalysts with “narrow‐bandgap” constraints.

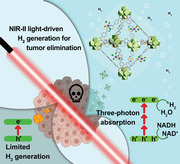

## 1 Introduction

Hydrogen (H_2_), a kind of clean/safe therapeutic molecule, has attracted widespread attention due to its outstanding antioxidant and anti‐cancer properties.^[^ ^1^ ^]^ However, the low solubility and easy diffusion of H_2_ pose challenges to the current conventional methods of hydrogen administration, such as inhalation of H_2_ or consumption of hydrogen‐rich water. These methods are inefficient and make it difficult to achieve sustained and controllable production of H_2_ at the site of the lesion, thus limiting the effectiveness of H_2_ in tumor treatment. To overcome this issue, one approach is to employ photocatalytic materials that utilize the reductive chemical substances present in the tumor microenvironment, such as glutathione and nicotinamide adenine dinucleotide phosphate (NAD(P)H), as sacrificial agents for artificial photocatalysts. This innovative method enables the continuous and controllable generation of H_2_ within tumor tissues through photocatalytic water splitting.^[^ ^2^ ^]^ Consequently, it offers a promising and emerging avenue for the controlled release and treatment of H_2_ in cancer therapy.^[^ ^3^ ^]^

Indeed, compared with visible light, near‐infrared (NIR) light possesses superior tissue penetration capabilities. By utilizing NIR light‐responsive photocatalysts to drive hydrogen production through water splitting, a viable solution is provided for the controlled release of H_2_ within deep‐seated tumor sites. This development holds great significance for H_2_ therapy in the field of cancer treatment.^[^ ^4^ ^]^ However, it is challenging to achieve efficient redox reactions with narrow‐bandgap photocatalytic materials due to their low redox potentials and high charge carrier recombination rates, which poses a significant challenge in the development of materials for NIR light‐driven hydrogen production.^[^ ^5^ ^]^ To overcome the challenge of low‐energy excitation states in NIR light that hinder effective redox reactions, researchers have proposed a strategy that involves utilizing photocatalysts capable of absorbing two or more NIR photons to reach a higher energy excited state. This approach enables efficient NIR photocatalysis, addressing the limitations posed by the low energy of NIR light and facilitating effective redox reactions.^[^ ^6^ ^]^ Multiphoton absorption materials offer an opportunity to overcome the limitations of traditional “narrow‐bandgap” photocatalysts used in NIR photocatalysis.^[^ ^7^ ^]^ These materials allow for sensitization to NIR light and provide a platform for the development of new and efficient NIR photocatalytic materials for hydrogen production. Additionally, their nonlinear optical properties enable precise activation of photocatalysts distributed in three‐dimensional space within tumor tissues through multiphoton excitation.^[^ ^8^ ^]^ This capability provides strong support for efficient NIR photocatalysis in H_2_ therapy, allowing for targeted activation and enhanced performance. Therefore, the fabrication of NIR photocatalytic materials with exceptional multiphoton absorption effects is highly important. This approach involves expanding and enhancing the NIR light absorption capacity of photocatalysts while achieving high electron–hole separation efficiency and utilization. This advancement holds crucial for the effective generation and high‐concentration accumulation of H_2_ in deep‐seated tumor tissues, thereby enhancing the therapeutic effectiveness of H_2_ treatment.

**Figure 1:**
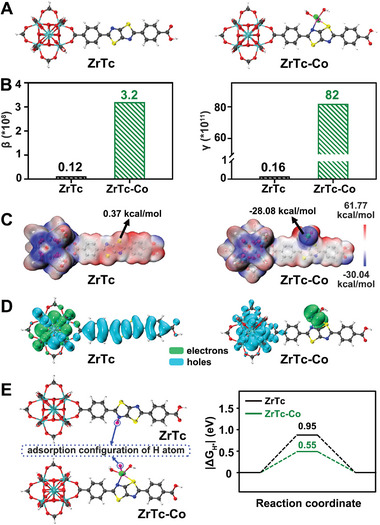
A) Calculated models of **ZrTc** and **ZrTc‐Co**. B) Comparison of the static first hyperpolarizability (β) and second hyperpolarizability (γ) between **ZrTc** and **ZrTc‐Co**. C) Electrostatic potential surface maps of **ZrTc** and **ZrTc‐Co**. D) Electron–hole distribution diagrams of **ZrTc** and **ZrTc‐Co**. Olive color: electrons; cyan color: holes. E) Adsorption configurations of H atoms and calculated Gibbs free energy profiles (∆*G*_H*_) of **ZrTc** and **ZrTc‐Co**.

Based on the research findings in the fields of tumor treatment,^[^ ^9^ ^]^ photocatalysis,^[^ ^10^ ^]^ and multiphoton material construction using metal‐organic frameworks (MOFs),^[^ ^11^ ^]^ it can be strongly argued that the fabrication of multiphoton responsive NIR photocatalysts based on MOFs for H_2_ therapy is theoretically feasible and has unique advantages. Herein, building upon our previous work on multiphoton responsive MOF (ZrTc),^[^ ^12^ ^]^ we introduced photocatalytically active sites of Co^II^ through post‐synthetic modification (termed ZrTc‐Co) to fabricate multiphoton responsive NIR photocatalytic materials for hydrogen production. Considering the coordination environment of Co^II^ and similar literature reports,^[^ ^13^ ^]^ we constructed an optimized model using Gaussian 16 (**Figure** **1**A). Based on this model, we calculated the relevant parameters for multiphoton and catalytic performance. As revealed by density functional theory (DFT) calculations, ZrTc‐Co exhibits larger static first hyperpolarizability (β) and static second hyperpolarizability (γ) than ZrTc, indicating its enhanced multiphoton absorption activity (Figure 1B). Furthermore, we evaluated the efficiency of photogenerated electron and hole separation, which is crucial for photocatalysis, using electrostatic potential and charge density distribution calculations.^[^ ^14^ ^]^ As depicted in Figure 1C, the average electro‐static potential around the Co^II^ site in ZrTc‐Co (−28.08 kcal mol^−1^) is more negative than that of ZrTc (0.37 kcal mol^−1^), signifying charge accumulation near the Co atom and effective separation of photogenerated charge carriers. This is further supported by the simulated separation of electrons and holes within ZrTc‐Co, indicating improved charge separation and favorable conditions for the hydrogen evolution reaction (HER) (Figure 1D). Additionally, the Gibbs free energy (Δ*G*_H*_) of the intermediate state in the HER process reflects the adsorption/desorption capacity of H^*^, which is a critical indicator of HER performance. Figure 1E illustrates the theoretically constructed adsorption configuration of H atoms, showing that the introduction of the Co^II^ active site leads to a closer thermoneutral state (|∆*G*_H*_|→0), facilitating the kinetics of the HER process.^[^ ^15^ ^]^ Taken together, these computational findings highlight that the incorporation of active Co^II^ sites within ZrTc enhances the multiphoton absorption coefficient and electron–hole separation, making ZrTc‐Co a promising candidate for multiphoton photocatalytic hydrogen evolution.

## 2 Results and Discussion

**Figure 2:**
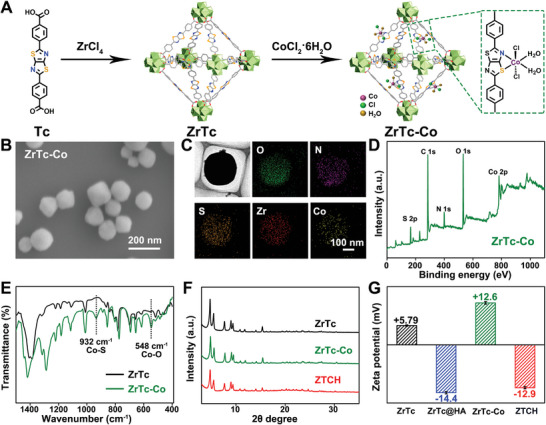
A) Synthesis routes of **ZrTc‐Co**. B) SEM image of **ZrTc‐Co** (scale bar: 200 nm). C) TEM image (lacey support film) and corresponding elemental mapping images of **ZrTc‐Co** (scale bar: 100 nm). D) XPS spectra of **ZrTc‐Co**. E) FT‐IR spectra of **ZrTc** and **ZrTc‐Co**. F) PXRD patterns of **ZrTc**, **ZrTc‐Co**, and **ZTCH** and the Lebail refinement of **ZrTc** and **ZrTc‐Co** are shown in Figure S6 (Supporting Information). G) Zeta potentials of **ZrTc**, **ZrTc@HA**, **ZrTc‐Co**, and **ZTCH** in deionized water (*n* = 5 independent experiments).

Motivated by the encouraging computational results and the potential for further biological applications, we embarked on the synthesis of nanoscale **ZrTc‐Co**. Initially, nanoscale **ZrTc** was prepared following our previous methodology with minor modifications.^[^ ^12^ ^]^ Subsequently, **ZrTc‐Co** was obtained by coordinating **ZrTc** with CoCl_2_•6H_2_O, as depicted in **Figure** **2**A. Scanning electron microscopy (SEM) and transmission electron microscopy (TEM) analyses revealed the octahedral morphology of **ZrTc‐Co**, while elemental mapping demonstrated the uniform distribution of cobalt (Figure 2B,C). Additionally, X‐ray photoelectron spectroscopy (XPS) results (Figure 2D; Figure S1, Supporting Information) confirmed the successful coordination of Co with **ZrTc**, with cobalt exhibiting a +2 oxidation state. The redshift observed in the binding energy of N 1s and S 2p for **ZrTc‐Co** compared to **ZrTc** indicated the coordination between Co and N/S atoms (Figure S2, Supporting Information). Furthermore, Fourier transform infrared (FT‐IR) spectroscopy provided additional evidence for the successful formation of **ZrTc‐Co**. Notably, the characteristic vibration peaks at 355, 548, and 932 cm^−1^ corresponded to the stretching modes of the Co─N, Co─O, and Co─S bonds, respectively (Figure 2E, Figure S3, Supporting Information).^[^ ^16^ ^]^ Combined with the aforementioned results and the mass spectra results of **ZrTc‐Co** (Figure S4, Supporting Information), the coordination mode of Co^II^ was confirmed, as depicted in Figure 2A, corroborating the findings from DFT calculations.

Subsequently, hyaluronic acid (HA) was used to wrap **ZrTc‐Co**, resulting in enhanced colloidal stability and cancer cell‐specific targeting ability (referred to as **ZTCH**, Figure S5, Supporting Information). PXRD patterns and SEM images revealed that the structure remained stable after the reaction (Figure 2F, Figures S6 and S7, Supporting Information). Furthermore, the introduction of Co^II^ led to a zeta potential of +12.6 mV for **ZrTc‐Co**, which shifted to a negative value (−12.9 mV) after HA coating (Figure 2G), indicating successful HA coating. Moreover, dynamic light scattering measurements of **ZTCH** in phosphate‐buffered saline (PBS) solution (pH 7.4) over a period of 7 days revealed an average diameter of 210 ± 10 nm with a low polydispersity index of 0.06 (Figure S8, Supporting Information), indicating good stability with negligible changes.

**Figure 3:**
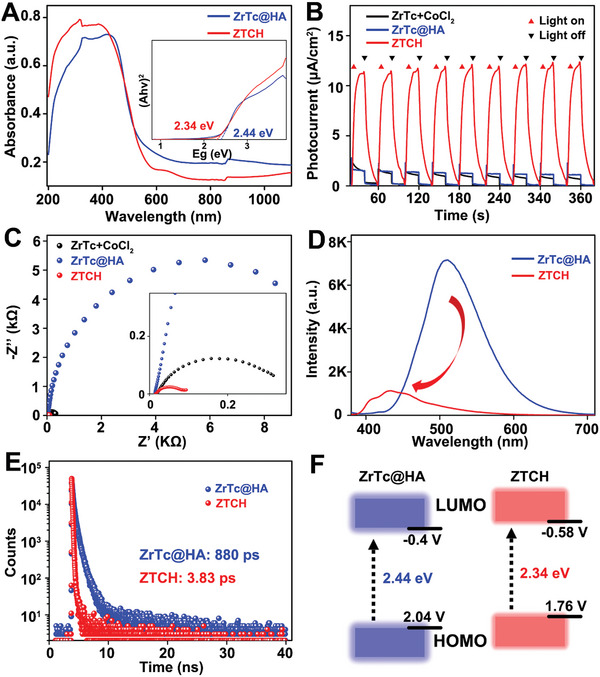
A) Diffuse reflectance ultraviolet–visible (DR UV–vis) spectra of **ZrTc@HA** and **ZTCH**. Inset: tauc plots of **ZrTc@HA** and **ZTCH**. B) Photocurrent response of **ZrTc@HA** and **ZTCH** under visible light irradiation. C) Electrochemical impedance spectroscopy Nyquist plots of **ZrTc@HA** and **ZTCH**. D) Room‐temperature PL emission spectra of **ZrTc@HA** and **ZTCH** (excitation wavelength: 370 nm). E) PL decay traces of **ZrTc@HA** and **ZTCH**. F) Energy level diagrams of **ZrTc@HA** and **ZTCH**.

Clearly, both **ZTCH** and the reference sample **ZrTc@HA** exhibit a prominent absorption band in the visible light region (**Figure** **3**A). Then, we conducted photoelectrochemical assays on the relevant samples. As illustrated in Figure 3B, the photocurrents of the **ZTCH** were significantly enhanced compared to pristine **ZrTc@HA** and the mixture of **ZrTc** with CoCl_2_•6H_2_O. This enhancement suggests that the introduction of Co^II^ facilitates the efficient separation of photogenerated electron–hole pairs. This finding is further supported by the results of electrochemical impedance spectroscopy (EIS) shown in Figure 3C, where **ZTCH** exhibits a notably smaller radius, indicating lower charge‐transfer resistance. In addition, the photoluminescence (PL) intensity of **ZTCH** clearly decreased, providing further evidence for the inhibition of electron–hole recombination (Figure 3D). Moreover, the PL lifetimes were determined to be 880 ps for **ZrTc@HA** and 3.83 ps for **ZTCH**, respectively, through fitting the time‐resolved PL spectra (Figure 3E). The shorter PL lifetime signifies faster transfer of photogenerated electrons and strongly inhibits photogenerated carrier recombination, suggesting higher photocatalytic activity. Furthermore, the optical bandgap of **ZrTc@HA** and **ZTCH** were calculated based on diffuse reflectance ultraviolet‐visible (DR UV–vis) spectroscopy, as shown in Figure 3F. **ZTCH** exhibited a decreased optical gap from 2.44 to 2.34 eV compared to that of **ZrTc@HA**. In addition, the conduction band positions of **ZrTc@HA** and **ZTCH** were determined to be −0.4 and −0.58 V (vs NHE, pH = 7) through Mott–Schottky measurements (Figure S9, Supporting Information). The corresponding energy level diagrams are presented in Figure 3F. The lowest unoccupied molecular orbital (LUMO) position of **ZTCH** is notably higher than that of **ZrTc@HA**, indicating stronger thermodynamic driving forces and electron reduction ability, which promote the photocatalytic hydrogen evolution capacity. Moreover, the LUMO levels of **ZTCH** is more negative than the H^+^/H_2_ redox potential (0.00 V vs NHE at pH = 7), indicating the capability for photocatalytic H_2_ evolution.^[^ ^17^ ^]^

**Figure 4:**
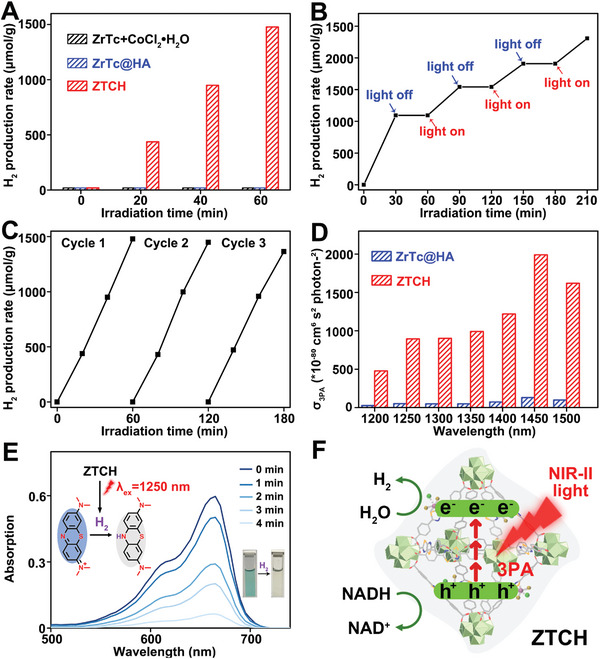
A) H_2_ production rates of **ZrTc** and CoCl_2_•6H_2_O physical mixture, **ZrTc@HA** and **ZTCH**. B) The switch off–on experiment of the **ZTCH**. C) Cycle experiment for H_2_ evolution of the **ZTCH**. The reaction conditions for the H_2_ generation experiments: 10 mg of catalyst, 30 mL of H_2_O, 0.4 mm NADH, 300 W xenon lamp using a *λ* > 400 nm cutoff filter. D) Three‐photon absorption cross section of **ZrTc@HA** and **ZTCH** (concentration: 500 µg mL^−1^, *λ*_ex_: 1200–1500 nm, laser power: 1 W cm^−2^, solvent: deionized water). E) Absorption spectrum of MB under 1250 nm laser irradiation within 4 min (laser power: 1 W cm^−2^). F) Schematic diagram of multiphoton photocatalytic hydrogen generation of **ZTCH**.

Encouraged by these promising findings, we investigated the photocatalytic water splitting performance of **ZTCH**, **ZrTc@HA**, and the physical mixture of **ZrTc** with CoCl_2_•6H_2_O under visible light irradiation in water. In addition, we performed an optimization process to determine the optimal Co^II^ content of **ZTCH** for efficient H_2_ generation (Figures S10 and S11, Supporting Information, detailed discussions are provided in Section S2.4, Supporting Information). To simulate the tumor microenvironment, nicotinamide adenine dinucleotide (NADH), which is a reductive substance overexpressed in tumors, was used as a sacrificial agent for holes. After 60 min of light irradiation, **ZTCH** with a Co content of 3.44% exhibited the highest H_2_ production rate, reaching 1477 µmol g^−1^, surpassing the performance of **ZrTc@HA** and the physical mixture of **ZrTc** with CoCl_2_•6H_2_O (**Figure** **4**A). Moreover, the consumption of NADH during the catalytic process was verified using UV–vis absorption spectroscopy. As depicted in Figure S12 (Supporting Information), it was evident that the characteristic peak of NADH at 340 nm gradually decreased, while the intensity of the absorption peak of NAD^+^ at 260 nm increased with prolonged irradiation time, indicating the consumption of NADH.^[^ ^18^ ^]^ Furthermore, a switch on/off experiment demonstrated the high controllability and stability of **ZTCH** for light‐responsive hydrogen generation (Figure 4B). Additionally, recycling experiments indicated no significant degradation in the hydrogen production rate over three consecutive catalytic runs, highlighting the excellent stability of **ZTCH** (Figure 4C). The SEM images taken after the catalytic process confirmed the structural integrity of the **ZTCH**, while the PXRD and XPS analyses showed negligible changes in the crystalline phase and oxidation state, respectively, further indicating the good stability of the **ZTCH** (Figure S13, Supporting Information). These results clearly demonstrate the high efficiency of **ZTCH** for hydrogen generation and establish it as an excellent photocatalyst.

To further explore the potential application of **ZTCH** in hydrogen therapy, it is crucial to evaluate its photocatalytic water splitting performance under NIR light, which holds significance. Building upon our previous research on the multiphoton‐excited fluorescence performance of **ZrTc**, we aimed to investigate the multiphoton activity of **ZTCH** under NIR‐II laser irradiation (1200–1500 nm) to evaluate its capability for NIR‐II‐induced hydrogen evolution. As illustrated in Figures S14 and S15 (Supporting Information), both **ZrTc@HA** and **ZTCH** exhibited significant three‐photon excited fluorescence. Moreover, the calculated three‐photon absorption cross section for **ZTCH** (8.95 × 10^−78^ cm^6^ s^2^ photon^−2^) was higher compared to that of **ZrTc@HA** (0.525 × 10^−78^ cm^6^ s^2^ photon^−2^) at 1250 nm, indicating its favorable NIR‐II photoexcitation ability (Figure 4D). Considering the excellent light‐activated H_2_ evolution capability of **ZTCH** under visible light and its impressive multiphoton absorption activity under NIR‐II laser irradiation, we further investigated its H_2_ evolution capacity under 1250 nm laser irradiation using methyl blue (MB) as a H_2_ probe, along with a synthesized hydrogen probe (NDO‐N_3_, Figure S16, Supporting Information).^[^ ^19^ ^]^ The reaction conditions for this investigation are described in the supporting information. Obviously, the intensity of the fluorescence emission at ≈580 nm from the NDO‐N_3_ probe significantly increased with light irradiation (Figure S17, Supporting Information), indicating the reduction of the azide group by H_2_ and confirming the effective light‐driven H_2_ generation of **ZTCH**. In addition, MB can be reduced to colorless leuco‐methylene blue by H_2_. As depicted in Figure 4E, the absorption at 665 nm of MB gradually decreased under 1250 nm laser irradiation, indicating the generation of H_2_. By employing the Lambert‒Beer law, the amount of H_2_ generated was calculated to be 21.54 µm under 1250 nm laser irradiation for 4 min (Figure S18, Supporting Information). Such high H_2_ production under NIR‐II light irradiation (Section S2.5, Supporting Information, Table S1, Supporting Information) lays the foundation for hydrogen therapy.^[^ ^20^ ^]^

Building upon the exceptional NIR‐II light‐driven multiphoton photocatalytic hydrogen generation ability of **ZTCH** (Figure 4F), we conducted further investigations into its antitumor performance both in vitro and in vivo. Leveraging the modification of HA, we initially explored the cellular uptake and targeting behavior of **ZTCH**. As depicted in Figure S19 (Supporting Information), **ZTCH** exhibited selective targeting toward HepG2 tumor cells (human hepatoma cells; CD44 receptor‐positive cells) while showing minimal uptake by Hek 293T cells (human embryonic kidney cells; CD44‐negative cells). Intracellular fluorescence of **ZTCH** was significantly observed in HepG2 cells, indicating successful cellular uptake and localization. This finding was further supported by the observed upregulation of CD44 expression in HepG2 cells following incubation with **ZTCH**, as demonstrated by Western blot assay (Figure S20, Supporting Information).^[^ ^21^ ^]^ Moreover, the mechanism of the cellular uptake pathway of **ZTCH** in HepG2 cells was explored, and the results demonstrated that the process was primarily dependent on energy‐dependent endocytosis and clathrin‐mediated endocytosis (Figure S21, Supporting Information).^[^ ^22^ ^]^

**Figure 5:**
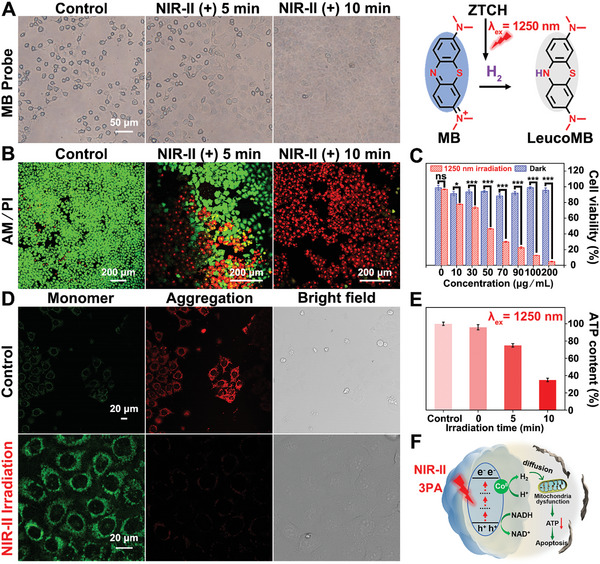
A) Qualitative analysis of H_2_ generation in MB‐stained Hep G2 cells under different irradiation time (*λ*_ex_ = 1250 nm, laser power: 0.1 W cm^−2^). B) CLSM images of HepG2 cells treated with **ZTCH** (50 µg mL^−1^) under different irradiation time, stained with calcein AM/PI (*λ*_ex_ = 1250 nm, laser power: 0.1 W cm^−2^). C) Viability of HepG2 cells incubated with different concentrations (0, 10, 30, 50, 70, 90, 100, 200 µg mL^−1^) of **ZTCH** with and without 1250 nm laser irradiation (laser power: 0.1 W cm^−2^). The data are shown as mean values ± standard deviations (*n* = 5). Statistical analysis was conducted with unpaired *t*‐test analysis (ns, not significant, *p < 0.05, **p < 0.01, ***p < 0.001). D) CLSM images of JC‐1‐stained HepG2 cells before and after irradiation with a 1250 nm laser for 10 min (laser power: 0.1 W cm^−2^). E) The intracellular ATP levels in HepG2 cells incubated with **ZTCH** under laser irradiation for different time (*λ*_ex_ = 1250 nm, laser power: 0.1 W cm^−2^). F) The proposed mechanism for hydrogen therapy based on the **ZTCH** under an NIR‐II laser (laser power: 0.1 W cm^−2^).

To determine the role of hydrogen in tumor therapy, we first evaluated the photothermal properties of **ZTCH**. The infrared imaging results demonstrated negligible temperature changes of **ZTCH** under 1250 nm laser irradiation for 10 min, thereby excluding thermal effects during activation (Figure S22, Supporting Information). Subsequently, MB was utilized to analyze the intracellular H_2_ release of **ZTCH** triggered by NIR‐II light. As depicted in **Figure** **5**A, the cells exhibited a blue color after coincubation with MB, and a gradual fading of the blue color was observed upon irradiation, indicating H_2_ generation by **ZTCH** under NIR‐II light irradiation. Furthermore, we used NDO‐N_3_ to detect H_2_ generation in HepG2 cells under 1250 nm laser irradiation (Figure S23, Supporting Information). Upon irradiation, an increase in fluorescence intensity was observed, demonstrating the intracellular release of H_2_ by **ZTCH** triggered by NIR‐II light. The amount of hydrogen generated was quantified by measuring the absorbance of MB at 665 nm using a microplate reader. According to a standard curve, the hydrogen concentration was calculated to be 19.5 µm within 15 min (Figures S24 and S25, Supporting Information). Additionally, the consumption of NADH in tumor cells was determined by the noticeable decrease in absorbance at 340 nm (Figure S26, Supporting Information). Overall, the aforementioned results showcase the efficient photocatalytic hydrogen generation process of **ZTCH** within Hep G2 cells under NIR‐II laser activation.

Furthermore, **ZTCH**‐induced cell apoptosis under NIR‐II light irradiation was evaluated using calcein acetoxymethyl ester (calcein AM, green fluorescence, a live cell indicator) and propidium iodide (PI, red fluorescence, a dead cell indicator). As depicted in Figure 5B, a distinct red fluorescent signal from PI was observed to intensify with prolonged laser irradiation time, indicating the remarkable therapeutic efficacy of **ZTCH**. Moreover, as illustrated in Figure 5C, **ZTCH** exhibited relatively slight effect on cell viability (evaluated by the methyl thiazolyl tetrazolium assay), even at concentrations as high as 200 µg mL^−1^ under dark conditions, indicating high cytocompatibility. However, significant decreases in cell viability were observed upon 1250 nm laser irradiation. Therefore, in summary, **ZTCH** can effectively consume NADH within cancer cells under NIR‐II light irradiation, resulting in hydrogen generation and triggering cell apoptosis. Previous studies have extensively demonstrated that hydrogen can specifically target mitochondria and induce cell apoptosis by inhibiting adenosine triphosphate (ATP) synthesis and impairing mitochondrial function.^[^ ^23^ ^]^ The mitochondrial membrane potential (MMP) is a critical indicator of mitochondrial health, and the apoptosis process is often accompanied by a decrease in the MMP, which can be detected through color changes in the tetrechloro‐tetraethyl benzimidazol carbocyanine iodide (JC‐1) dye. As depicted in Figure 5D, the irradiation group exhibited prominent green fluorescence, indicating a low MMP and monomer formation, suggesting that the generated H_2_ effectively disrupted the MMP. Furthermore, the ATP content significantly decreased upon laser irradiation (Figure 5E). These compelling results collectively demonstrate that **ZTCH** can serve as a highly effective NIR‐II‐responsive photocatalyst, inducing cancer cell death through H_2_‐mediated mitochondrial damage (Figure 5F).

**Figure 6:**
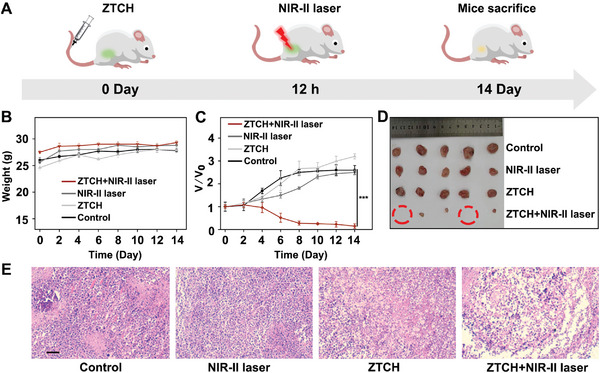
A) A schematic diagram illustrating the development and treatment process of the H22 subcutaneous tumor‐bearing female ICR mouse model. B) The body weights of ICR mice of the control, NIR‐II laser, **ZTCH**, and **ZTCH** with NIR‐II laser groups (*n* = 5 independent experiments). C) The tumor volumes of the control, NIR‐II laser, **ZTCH** and **ZTCH** with NIR‐II laser groups (*n* = 5 independent experiments). D) Photographs of the tumors in different treatment groups. E) H&E‐stained tumor sections from different treatment groups (scale bar: 500 µm). The data are shown as mean values ± standard deviations (*n* = 5). Statistical analysis was conducted with unpaired *t*‐test analysis (ns, not significant, *p < 0.05, **p < 0.01, ***p < 0.001).

In general, NIR light offers the advantage of deeper penetration, making it particularly beneficial for the treatment of deep‐seated tumors. To evaluate the penetration depth of **ZTCH** under 1250 nm NIR light, myocardial tissue sections from mice were obtained. As illustrated in Figure S27 (Supporting Information), the penetration depth reached at least 140 µm under 1250 nm irradiation. Additionally, the efficacy of **ZTCH** treatment in deep tumors was verified using Hep G2 3D multicellular spheroids (MCSs). The MCSs were treated with **ZTCH** and stained with calcein AM/PI (Figure S28, Supporting Information). In the 405 nm‐treated MCSs, red fluorescence from PI was observed only at the edge of the MCSs. In contrast, under 1250 nm excitation, the red fluorescence of PI was distributed throughout the tumor section, even in the tumor center, and was clearly visible. These findings indicate that **ZTCH** is an effective NIR light‐induced hydrogen therapy for the treatment of deep‐seated tumors. Therefore, we proceeded to evaluate the in vivo cancer targeting and therapeutic behavior of **ZTCH** using H22 cancer‐bearing mice (**Figure** **6**A). The excellent biosafety of **ZTCH** was confirmed based on its low hemolysis ratio (3.6%), which is generally considered safe for intravenous administration (Figure S29, Supporting Information). Furthermore, the tumor‐targeting behavior of **ZTCH** was assessed through fluorescence imaging (Figure S30, Supporting Information), which clearly indicated the accumulation of **ZTCH** at the tumor site 24 h after intravenous injection. Additionally, pharmacokinetic analysis revealed a relatively long blood circulation half‐time of 2.26 h (Figure S31, Supporting Information).

Considering these exceptional properties, we conducted further investigations to evaluate the anticancer efficacy of **ZTCH** under 1250 nm irradiation (Figure 6A). Tumor‐bearing mice were divided into control, NIR‐II laser irradiation, **ZTCH**, and **ZTCH** with NIR‐II laser irradiation groups. As shown in Figure 6B, Figures S32 and S33 (Supporting Information), all treatment groups did not show alterations in body weight, visible damage to major organs, or significant changes in hematological parameters after treatment. Furthermore, the experimental group clearly exhibited significant tumor inhibition (95%) after treatment, demonstrating the high efficacy of photocatalytic hydrogen therapy using **ZTCH** (Figure 6C,D, Figure S34, Supporting Information, Table S2, Supporting Information). Hematoxylin and eosin (H&E) staining of tumor sections revealed that **ZTCH**, when combined with laser irradiation, induced noticeable tumor cell apoptosis (Figure 6E). However, **ZrTc@HA** exhibited much lower in vitro and in vivo anticancer efficiency under the same conditions (Figures S35–S37, Supporting Information, Table S3, Supporting Information). Therefore, the remarkable NIR‐II light‐driven multiphoton photocatalytic hydrogen evolution ability of **ZTCH** makes it a promising candidate for tumor therapeutic applications.

## 3 Conclusion

In conclusion, a multiphoton responsive MOF, **ZTCH**, with a considerable multiphoton absorption cross‐section and electron–hole separation efficiency for NIR‐II light‐driven multiphoton photocatalytic H_2_ therapy. By utilizing excessive intracellular NADH as a sacrificial agent, **ZTCH** exhibits remarkable stability, enabling the generation of H_2_ both in aqueous solutions and in intracellular environments. Furthermore, the introduction of hyaluronic acid modification enhances the colloidal stability of **ZTCH** and endows it with cancer cell‐specific targeting capabilities, facilitating precise NIR‐II light‐triggered H_2_ generation at deep‐seated tumor sites for effective oncotherapy. This work provides an innovative approach for the controllable fabrication of multiphoton photocatalytic materials to overcome the limitations imposed by traditional near‐infrared photocatalysts with “narrow‐bandgap” constraints, which offers a promising route for enabling the controlled generation and high‐concentration release of H_2_ at deep tumors and improving H_2_ therapeutic outcomes.

## 4 Experimental Section

Materials and preparation of **ZTCH** are provided in Supporting Information.

### DFT Calculations

Geometry optimization was calculated at B3LYP hybrid functional with 6‐31G(d) basis sets in GAUSSIAN 16 package.^[^ ^24^ ^]^ The static hyperpolarizabilities was calculated by the sum‐over‐states (SOS) method using time‐dependent density functional theory (TD‐DFT).^[^ ^25^ ^]^ Multiwfn 3.8 was used to analyze molecular orbitals (MOs) and VMD 193 software was used for molecular visualization.^[^ ^26^ ^]^

### Photoelectrochemical Measurements

The CHI 760E electrochemical workstation from Chenhua Instrument was employed based on a standard three‐electrode system. A photocatalyst‐coated ITO (size: 20*10*1.1 mm; coated area: 10*10 mm) was used as working electrode, Pt plate is used as a counter electrode, and an Ag/AgCl electrode is used as a reference electrode for photocurrent measurement. 300 W Xenon lamp with *λ* > 400 nm cutoff filter was used as light source. The mixture of 2 mg catalysts and 200 µL Nafion solution was dispersed uniformly by ultrasonic and dropped on ITO. A 0.5 m Na_2_SO_4_ solution was used as the electrolyte. Then, the photoreactive signals were recorded under chopped light with at +0.4 V. The switch of the light is realized by a cardboard covered with tinfoil. EIS and Mott–Schottky plot measurements were carried out with the same as that in the photocurrent measurements. EIS was performed at 1.2 V in a frequency range from 1 to 10^5^ Hz.

### Three‐Photon Excited Fluorescence Spectroscopy and Three‐Photon Absorption Cross‐Section

Three‐photon excited fluorescence spectra were obtained through fluorescence contrast method using Coherent Astrella+TOPAS Prime (1150–2600 nm, 1 kHz, 120 fs) as the light source. And the Rhodamine 6G (0.479 mg mL^−1^) in ethanol is used as a reference sample. The concentration of **ZrTc@HA** and **ZTCH** was 500 µg mL^−1^. Additionally, the three‐photon absorption cross‐section was calculated according to the previous works.^[^ ^27^ ^]^

### Hydrogen Detection

Methylene blue (MB) can be rapidly reduced and discolored by hydrogen. Briefly, **ZTCH** (300 µg mL^−1^) was dispersed into 3 mL of deionized water with NADH (400 µm). Then, 30 µL of MB solution (1 mm)) was mixed in a sealed absorption vessel after purging with N_2_ adequately. Then, it was irradiated under 1250 nm (1 W cm^−2^) laser, and the absorption spectra of MB was collected every 1 min. The fluorescence detector (NDO‐N_3_) was synthesized according to the literature and its synthesize route and ^1^H‐NMR was shown in Figure S16 (Supporting Information). **ZTCH** (300 µg mL^−1^) was dispersed into 3 mL of deionized water with NADH (400 µm). Then, 30 µL of NDO‐N_3_ solution (1 mm)) was mixed in a sealed emission vessel after purging with N_2_ adequately. Then, it was irradiated under 1250 nm (1 W cm^−2^) laser, and the emission spectra was collected every 1 min.

### Western Blot Assay

HepG2 cells and Hek 293T cells were cultured in a 6 well plate and incubated for 24 h. HepG2 cells were then treated with HA (50 µg mL^−1^) and **ZTCH** (50 µg mL^−1^) separately for 12 h. Following PBS washing, HepG2 cells, HepG2 cells incubated with **ZTCH** and Hek 293T cells were lysed using loading buffer and heated at 100 °C for 5 min. The cell lysate was subjected to centrifugation at 10 000 rpm for 30 min. The resulting supernatant was collected and loaded onto a 15% SDS‐PAGE gel. Subsequently, the proteins were transferred to NC membranes using an 80 V electric current for 90 min. The membrane was then washed three times with TBST and blocked with skim milk powder at room temperature for 1 h. For antibody incubation, a primary CD44 antibody, diluted at 1:1000 in the blocking solution, was applied to the membrane and incubated overnight at 4 °C. After three 10‐min washes with TBST, a secondary antibody, diluted at 1:1000, was applied to the membrane for 1 h at room temperature. The membrane was once again washed three times with TBST for 10 min each. Finally, the expression of proteins was detected using a chemiluminescence ECL substrate.

### Cell Culture

The HepG2 cells were cultured in 25 cm^2^ culture flasks in DMEM at 37 °C in a CO_2_ incubator (95% relative humidity, 5% CO_2_). Additionally, the cells were supplemented with 10% fetal bovine serum, 100 units mL^−1^ penicillin and 50 units mL^−1^ streptomycin.

### Cytotoxicity Assays

HepG2 cells were cultured in 96‐well plate and incubated for 24 h. **ZTCH** solutions (0, 10, 30, 50, 70, 90, 100, 200 µg mL^−1^) were added into the culture medium and then incubated at 37 °C in 5% CO_2_ for 12 h. Removed the cell medium solutions and added 100 µL fresh medium mixed with MTT (5 mg mL^−1^) into each well. After incubating for 4 h, the MTT medium was removed and replaced with 100 µL DMSO. The absorbance at 490 nm was then measured using a microplate reader. The cell viability was calculated by comparing the absorbance of the experimental wells to that of the cell control wells.

### Live/Dead Assay with Calcein AM/PI

HepG2 cells were treated with **ZTCH** (50 µg mL^−1^) for 12 h. To assess cell viability, Calcein AM and PI stains were applied. Then, confocal laser scanning microscope was employed to collect fluorescence images following 1250 nm laser irradiation (0.1 W cm^−2^) for different time.

### Culture of 3D Multicellular Spheroids (3D MCSs)

In a 25 mL cell culture flask, 5 mL Poly HEMA solution was added and then ethanol was evaporated at 37 °C. Then the flask was sterilized for 24 h under ultraviolet lamp. Before 200 µL HepG2 cells suspension was added, the culture flask was washed with PBS for twice. 3D multicellular spheroids were formed with appropriate diameter after 3–5 days.

### Animal Experiments

The animal procedures conducted in this study were approved by the Institutional Animal Care and Use Committee (IACUC) of Anhui University (Approval number: IACUC(AHU)‐2024‐017), in accordance with the ethical guidelines outlined in the National Standard of China GB/T35892‐2018 for the Ethical Review of Experimental Animal Welfare. ICR mice (female, 18–22 g) were provided by the Zi Yuan Laboratory Animal Technology Co. Ltd. (Hangzhou, China). Significant efforts were made to minimize the use of animals and to alleviate their pain and discomfort. Female ICR mice with H22 tumor model were used for fluorescence imaging and antitumor test in vivo. The mice were treated via intratumorally injection with **ZTCH** (5 mg kg^−1^).

### In Vivo Fluorescence Imaging Performance of **ZTCH**

Female ICR mice bearing H22 tumor received intravenous injections of **ZTCH** solution (5 mg kg^−1^). To evaluate fluorescence imaging performance, images were obtained at 0, 2, 4, 6. 8. 10, 12, 24 h, respectively, using IVIS multimodal in vivo imaging system. And then, the mice were sacrificed humanely to obtain their major tissues and tumors and imaged by the imaging system.

### In Vivo Therapeutic Assessment

ICR female mice with H22 tumor were divided into four groups (*n* = 5): Control (PBS solution (pH = 7.4)), NIR‐II laser (1250 nm, 0.1 W cm^−2^, 5 min), **ZTCH** and **ZTCH** with NIR‐II laser (1250 nm, 0.1 W cm^−2^, 5 min). 5 mg kg^−1^ of **ZTCH** were intravenous injected into the mice. The tumor size and mice weight were recorded every two days. Mice were humanely sacrificed for further histopathological analysis after 14 days.

### Statistical Analysis

The experiments were repeated at least three times and the results were presented as mean ± SD. Sample size (*n*) for each statistical analysis is provided in the respective figure legends. Statistical analyses were performed using the unpaired *t*‐test analysis. GraphPad Prism software was utilized for analyzing the date. The differences of all date were regarded as significant for p‐value: ns, not significant, *p < 0.05, **p < 0.01, ***p < 0.001.

## Conflict of Interest

The authors declare no conflict of interest.

## Supporting information

Supporting Information

## Data Availability

The data that support the findings of this study are available from the corresponding author upon reasonable request.
