# Supplementary material for: Harnessing Metal‐Organic Frameworks for NIR‐II Light‐Driven Multiphoton Photocatalytic Water Splitting in Hydrogen Therapy
Source: Adv Sci (Weinh). 2024 Aug 9;11(38):2405643. doi: 10.1002/advs.202405643 (PMC11481200; doi:10.1002/advs.202405643)
Supplement: Supplementary file 1 — Supporting Information [file ADVS-11-2405643-s001.docx]

Supporting Information

Harnessing Metal-Organic Frameworks for NIR-II Light-Driven Multiphoton Photocatalytic Water Splitting in Hydrogen Therapy

Xin Lu,^‡^ Xinlei Yu,^‡^ Bo Li,^‡^ Xianshun Sun^*^, Longjiu Cheng, YuanZhong Kai, Hongping Zhou, Yupeng Tian, Dandan Li^*^

**1. Materials and Apparatuses**

All materials were obtained from commercial supplies and used without further purification unless noted otherwise. PXRD patterns were recorded on SmartLab 9KW. The absorption spectra were recorded on a PerkinElmer Lambda 1050+UV/Vis/NIR spectrophotometer. The emission spectra were performed on a Hitachi F-7010 fluorescence spectrophotometer. SEM was carried out on a REGULUS8230*. TEM was carried out on a JEM-2100. Horiba Fluoro max plus was used to detect fluorescence lifetime. The H_2_ production experiments were obtained by gas chromatography (Panon A910 plus). One-photon and multi-photon fluorescence imaging data were collected using Lecia STELLARIS 8, which equipped with femtosecond laser (wavelength: 680 - 1300 nm, 80 MHz, 140 fs).

**2. Supporting Experimental Information**

**2.1 Synthesis of ZrTc**

The dicarboxylic acid ligand, **Tc**, was synthesized according to our previous work.^[1]^

**Tc** (0.014 mol/L), ZrCl_4_ (0.01 mol/L) and benzoic acid (0.14 g) were dissolved in 30 mL DMF, then added to a 50 mL of Teflon-lined stainless-steel autoclave (Anhui Kemi Instrument Co., Ltd), and heated at 120°C for 72 h. The solid was centrifugation and washed with DMF and ethanol for three times, then dried in a vacuum at 60°C to get yellow powder (yield: 60.6%).

**2.2 Synthesis of ZrTc-Co**

CoCl^­^_2_·6H_2_O (5/20/50 mg) and 5 mg of **ZrTc** were dispersed in 50 mL DMF, and the mixture was stirred at 80°C for 24 h to get **ZrTc-Co (1.45%)**, **ZrTc-Co (3.44%)** and **ZrTc-Co (4.75%)**, respectively (yield: 90.2%, 89.3%, 87.9%). The product was obtained after centrifugation, and washed with DMF, ethanol, and deionized water, then dried under freeze drying.

**2.3 Synthesis of ZTCH**

Hyaluronic acid (HA, 50 mg) was dispersed in deionized water under sonication, then **ZrTc-Co** (2 mg) was added and stirred at room temperature overnight. The solid was collected by centrifugation and washed with deionized water for three times, then dried under freeze drying.

**2.4 Optimal process for Co(II) contents**

The samples (**ZrTc-Co**) with different Co contents (1.45%, 3.44% and 4.75%) have been synthesized. Obviously, **ZrTc-Co** with a Co content of 3.44% exhibited the highest photocurrent and the lowest charge-transfer resistance (Figure S10). Moreover, it displayed the highest H_2_ production (1477 μmol/g) compared with other samples (**ZrTc-Co** (1.45%): 505 μmol/g, **ZrTc-Co** (4.75%):1103 μmol/g) due to the introduction of excessive active site (Co) may become an electron trap and reduce the photocatalysis performance (Figure S11).^[2]^ Therefore, the sample with a Co content of 3.44% (Zr content: 8.5%) was selected and presented in the main text.

**2.5 Discussions on the uniqueness of ZTCH for NIR-driven photocatalytic hydrogen production**

Firstly, the construction of near-infrared (NIR) light-activated hydrogen production materials that enable controlled generation and high-concentration release of H_2_ in deep tumors is of significant scientific importance. However, it is challenging to achieve efficient redox reactions with narrow-bandgap photocatalytic materials due to their low redox potentials and high charge carrier recombination rates. To date, very limited NIR-responsive hydrogen production materials for tumor elimination have been developed.^[3]^

Secondly, to address the key technical challenge of low efficiency in oxidation-reduction reactions for narrow-bandgap photocatalytic materials, this work provides an innovative approach of controllable fabrication of multiphoton photocatalytic materials to overcome the limitations imposed by traditional near-infrared photocatalysts with "narrow-bandgap" constraints.

Thirdly, the anticancer performance between **ZTCH** and other reported NIR responsive photocatalysts has been compared. It is worth noting that it is the first report on NIR-driven photocatalytic H_2_ production using multiphoton absorption materials. Moreover, **ZTCH** displays the longest excitation wavelength for H_2_ production. Besides, its H_2_ production activity is considerable compared with other ones (Table S1).

Last but not least, herein, it was employed as a research model to fabricate NIR-driven multiphoton photocatalytic H_2_ evolution for tumor elimination. Given the tailorability and great diversity of MOFs, the fabrication concept for this type of photocatalyst holds great promise for enhanced H_2_ therapy efficiency in future endeavor.


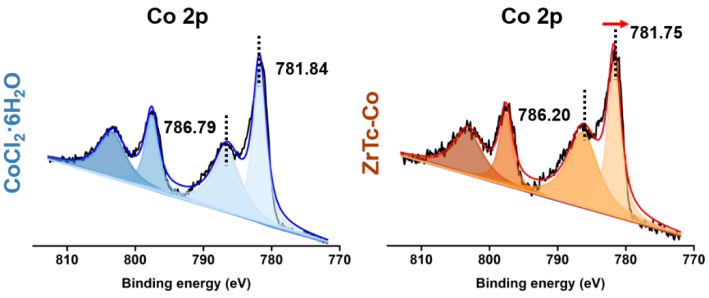


**Figure S1.** Co 2p XPS spectra of the CoCl_2_·6H_2_O and **ZrTc-Co**.


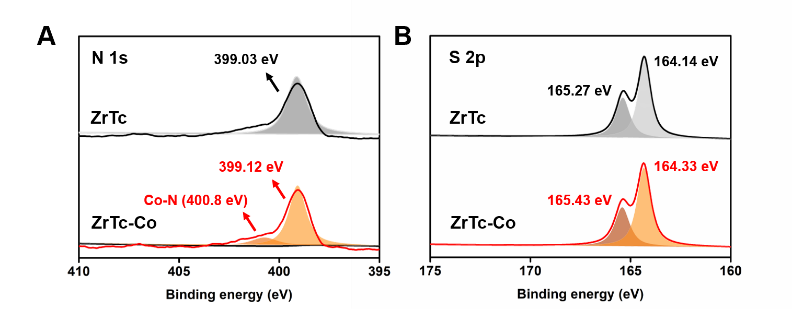


**Figure S2.** (A) N 1s and (B) S 2p XPS spectra of the **ZrTc** and **ZrTc-Co**.


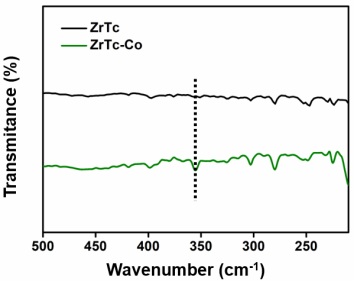


**Figure S3.** The far-infrared spectrum of **ZrTc** and **ZrTc-Co**.


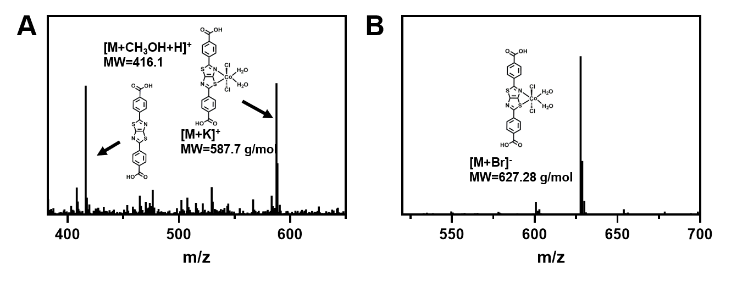


**Figure S4.** The ESI-MS spectra of **ZrTc-Co** in (A) positive and (B) negative mode. **ZrTc-Co** was decomposed in nitric acid (65 - 68 %) and the solid was obtained by centrifugation and dissolved in methanol.


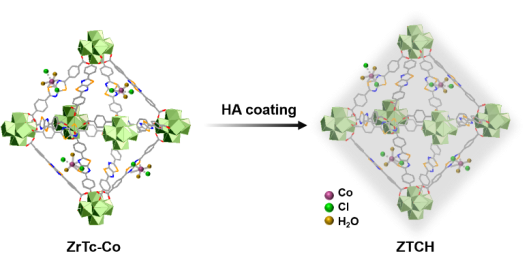


**Figure S5.** Schematic diagram of synthesis route for **ZTCH**.


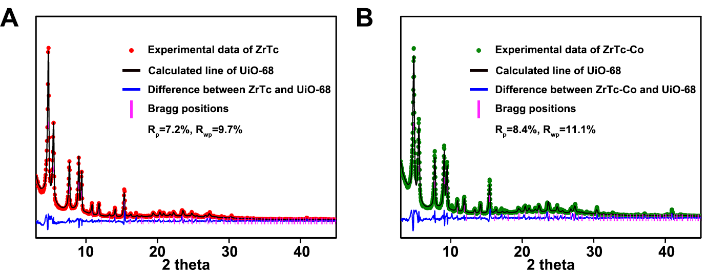


**Figure S6.** Refined results of the PXRD data of (A) **ZrTc** and (B) **ZrTc-Co**.


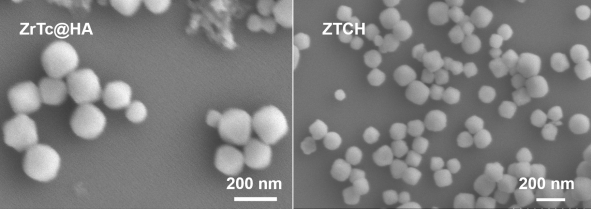


**Figure S7.** SEM image of **ZrTc@HA** and **ZTCH**.


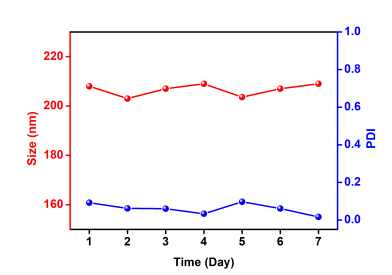


**Figure S8.** Particle size distribution and PDI changes of **ZTCH** in PBS solution (pH 7.4) during 7 days.


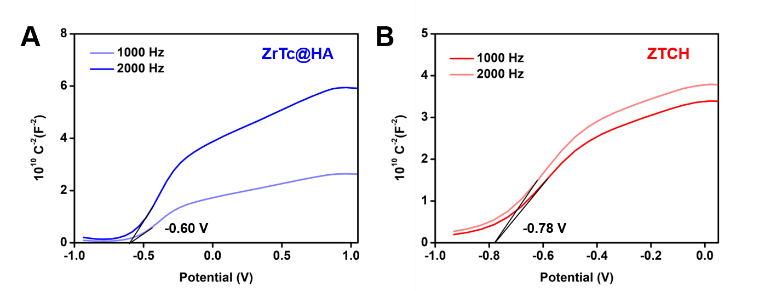


**Figure S9.** Mott-Schottky measurements of (A) **ZrTc@HA** and (B) **ZTCH**.


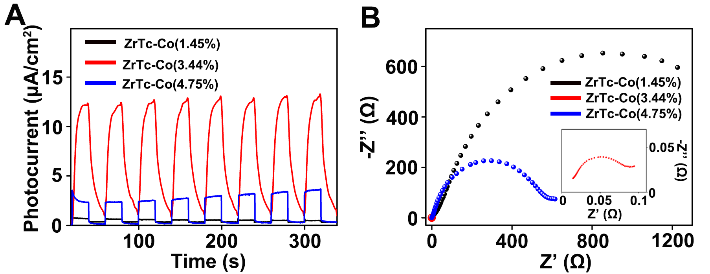


**Figure S10.** (A) Photocurrent response of **ZrTc-Co** (1.45%), **ZrTc-Co** (3.44%) and **ZrTc-Co** (4.75%) under visible light irradiation. (B) Electrochemical impedance spectroscopy Nyquist plots of **ZrTc-Co** (1.45%), **ZrTc-Co** (3.44%) and **ZrTc-Co** (4.75%). Insert: enlarged electrochemical impedance spectroscopy Nyquist plots of **ZrTc-Co** (3.44%).


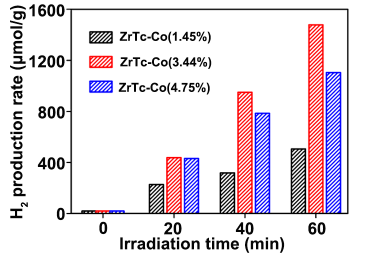


**Figure S11.** H_2_ production rate of **ZrTc-Co** (1.45%), **ZrTc-Co** (3.44%) and **ZrTc-Co** (4.75%) (10 mg catalyst, 30 mL of H_2_O, 0.4 mM NADH, 300 W xenon lamp using λ > 400 nm cutoff filter).


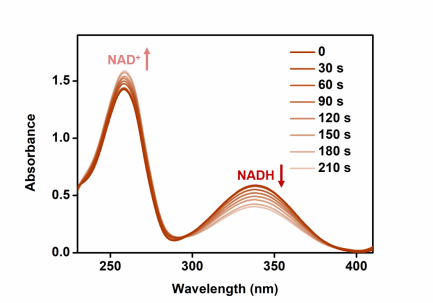


**Figure S12.** Absorption spectra of NADH under light irradiation for different time (concentration of **ZTCH**: 300 μg/mL; 0.4 mM NADH; 300 W xenon lamp using λ > 400 nm cutoff filter).


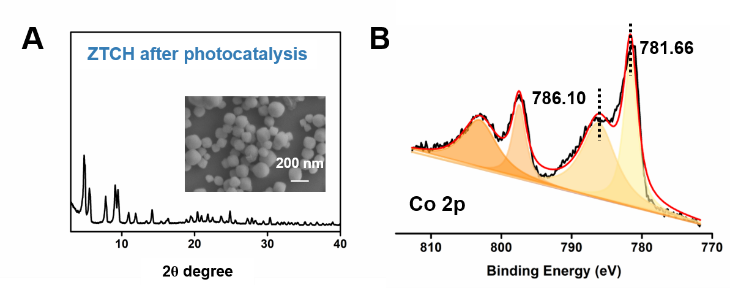


**Figure S13.** (A) PXRD spectra of **ZTCH** after photocatalysis process. Inset: SEM image of **ZTCH** after photocatalysis process. (B) XPS spectra of **ZTCH** after photocatalysis process.


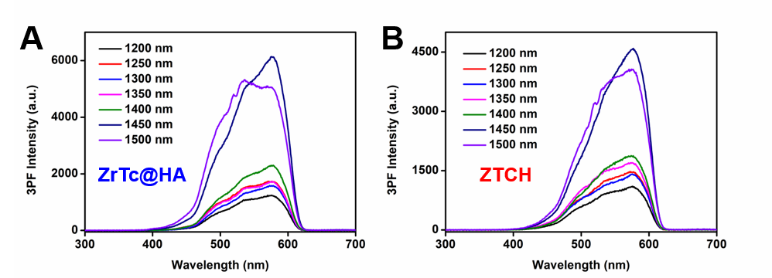


**Figure S14.** Three photon excited fluorescence spectra of (A) **ZrTc@HA** and (B) **ZTCH** (concentration: 500 μg/mL; solvent: deionized water; excited wavelength: 1200 - 1500 nm; laser power: 1 W/cm^2^).


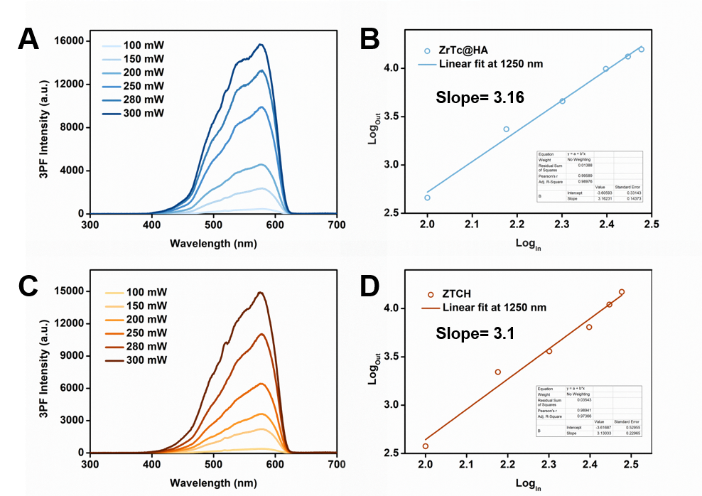


**Figure S15.** (A) Three photon excited fluorescence spectra and (B) three-photon verification of **ZrTc@HA** under different laser power. (C) Three-photon fluorescence spectra and (D) three-photon verification of **ZTCH** under different laser power (concentration: 500 μg/mL; solvent: deionized water; excited wavelength: 1250 nm).


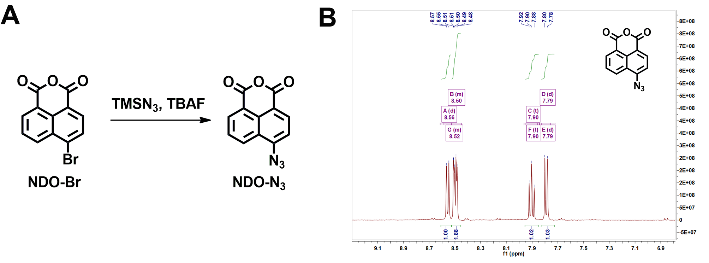


**Figure S16.** (A) Synthesized route of NDO-N_3_. (B) ^1^H-NMR spectra of NDO-N_3_ (400 MHz, *d_6_*-DMSO).


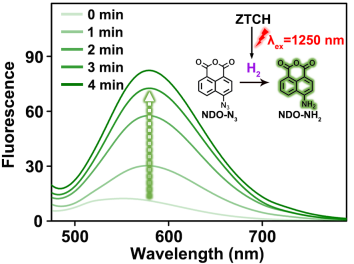


**Figure S17.** Fluorescence spectra of NDO-N_3_ under 1250 nm laser irradiation within 4 min (laser power: 1 W/cm^2^).


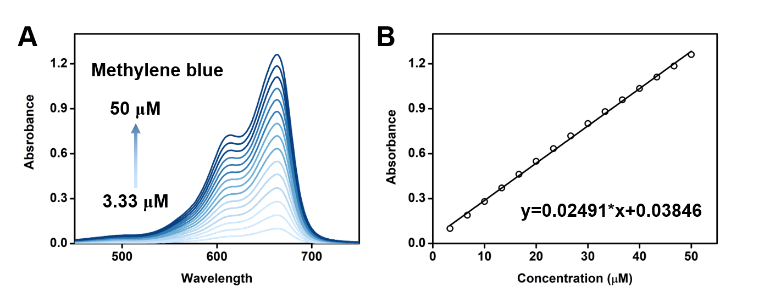


**Figure S18.** (A) The absorption spectra of MB with different concentration (3.33 μM - 50 μM). (B) Linear fit of concentration and absorbance.


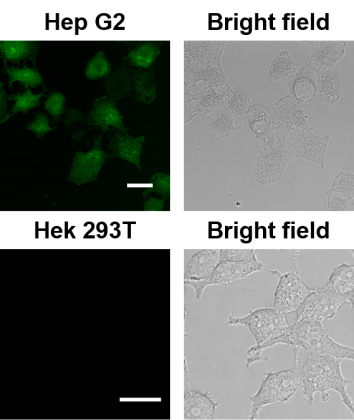


**Figure S19.** Confocal images of **ZTCH** incubated with HepG2 cells and Hek 293T cells (scale bar: 20 μm).


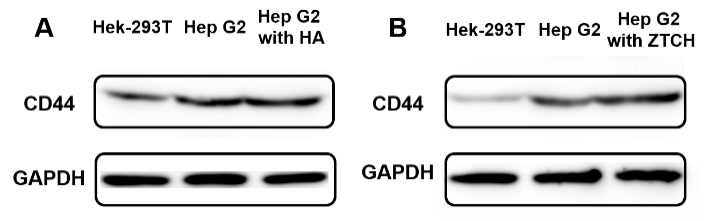


**Figure S20.** Western blot assay of CD44 in Hek-293T cells, HepG2 cells and HepG2 cells incubated with (A) HA (50 μg/mL) and (B) **ZTCH** (50 μg/mL).
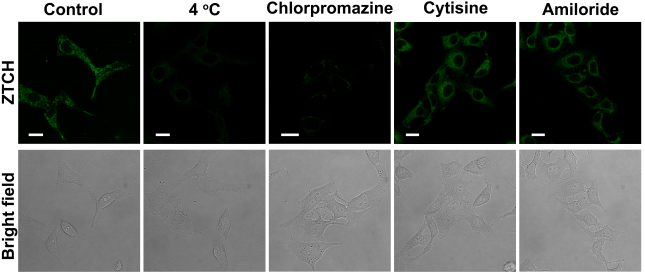


**Figure S21.** Confocal images of the HepG2 cells incubated with different endocytic inhibitors: low temperature (4°C); cytisine (+Cytisine, 200 μg/mL); CPZ (+Chlorpromazine, 80 μg/mL); andamiloride (+Amiloride, 200 μg/mL). Scale bar: 20 μm.


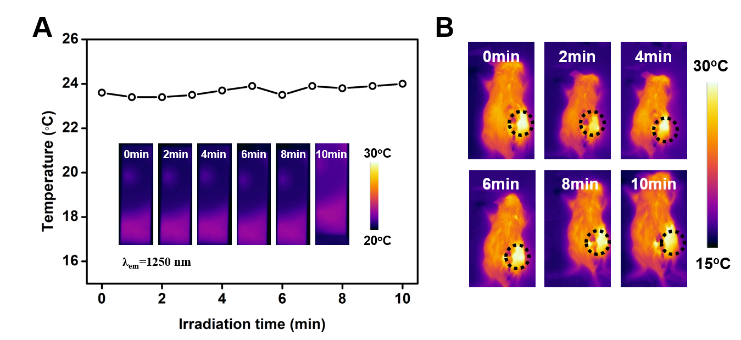


**Figure S22.** (A) Temperature changes of **ZTCH** under 1250 nm laser irradiation (laser power: 1 W/cm^2^) for 10 min, which was recorded by infrared imaging camera. (B) The photos of temperature in tumor site of **ZTCH** under 1250 nm laser irradiation (laser power: 0.1 W/cm^2^) using an infrared imaging camera. Concentration: 500 μg/mL; solvent: deionized water.


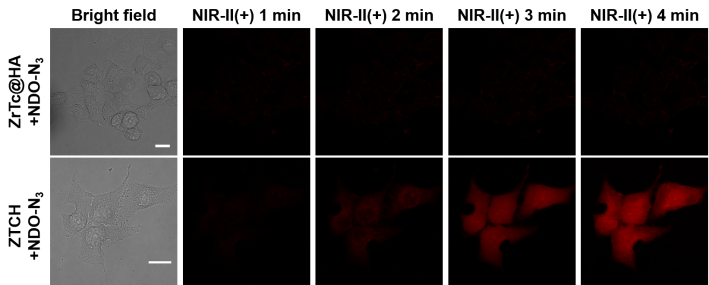


**Figure S23.** CLSM images of NDO-N_3_ in HepG2 cells under 1250 nm laser irradiation within 4 min (λ_ex_ = 440 nm, λ_em_ = 570-590 nm, laser power: 0.1 W/cm^2^, scale bar: 20 μm).





**Figure S24.** The relationship between absorbance at 665 nm and concentration of MB recorded on a microplate reader (concentration: 3.33 μM - 33.33 μM).





**Figure S25.** Concentration of H_2_ generation under 1250 nm laser irradiation for different time (0, 3, 6, 9, 12 and 15 min) calculated according to the standard curve recorded on microplate reader (cell: HepG2; concentration of **ZTCH**: 50 μg/mL; laser power: 0.1 W/cm^2^).


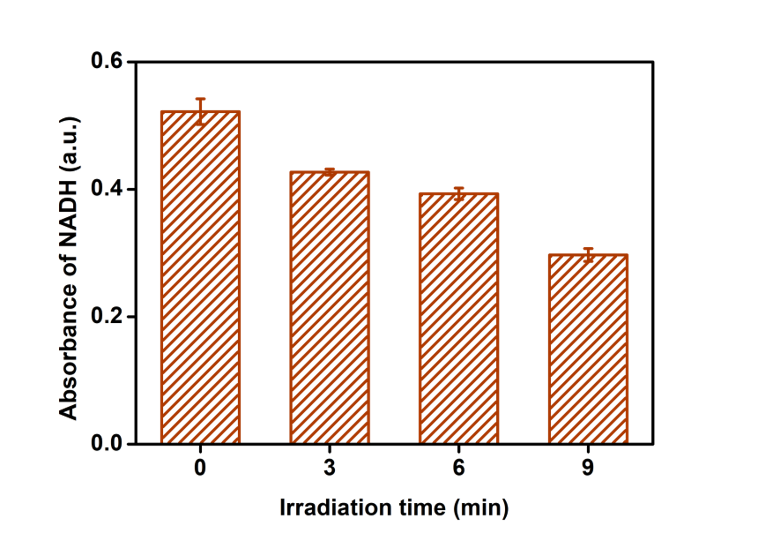


**Figure S26.** Absorbance of NADH at 340 nm of HepG2 cell treated with **ZTCH** and under 1250 nm laser irradiation for different time (0, 3, 6 and 9 min) recorded on microplate reader (concentration of **ZTCH**: 50 μg/mL; laser power: 0.1 W/cm^2^).


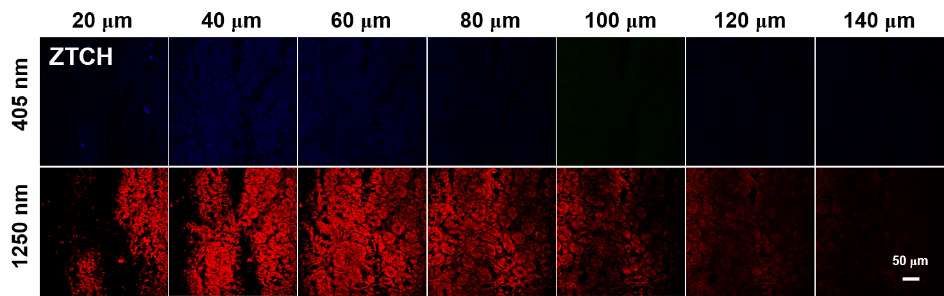


**Figure S27.** Fluorescence images of tissue section incubated with **ZTCH** (50 μg/mL) with different penetration depth along the z axis under 405 nm (0.1 W/cm^2^) and 1250 nm (0.1 W/cm^2^) laser irradiation.


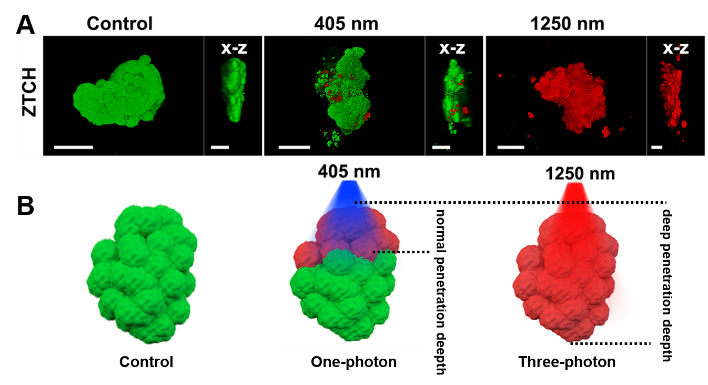


**Figure S28.** (A) HepG2 MCSs incubated with **ZTCH**, and incubated with calcein AM (indicator of living cells)/PI (indicator of dead cells). HepG2 MCSs were irradiated by 405 nm, and 1250 nm laser for 15 min (fixed power: 0.1 W/cm^2^, scale bar: 100 μm). (B) Schematic diagram of deep tumor elimination.


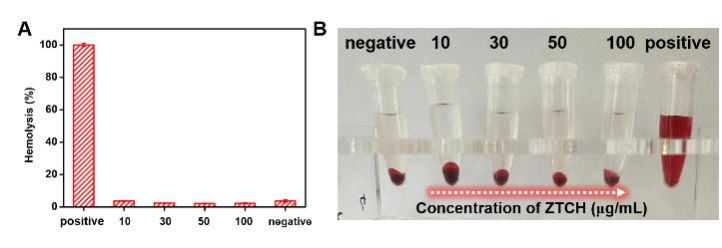


**Figure S29.** (A) Hemolysis ratio of red blood cells incubated with **ZTCH** (10, 30, 50, 100 µg/mL). (B) Image of red blood cells centrifugation and treatment with different concentrations of **ZTCH**.


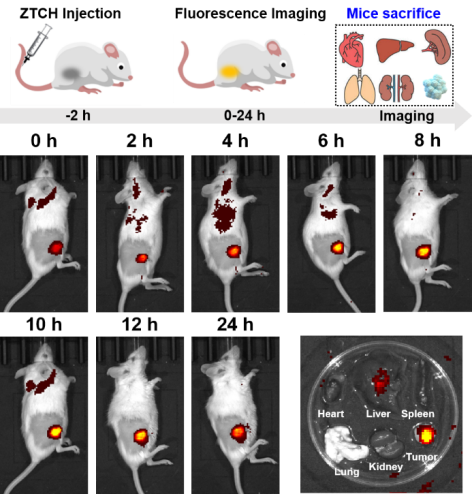


**Figure S30.** *In vivo* fluorescence images of tumors in mice and major organs after the intravenous injection of **ZTCH** within 24h.

**
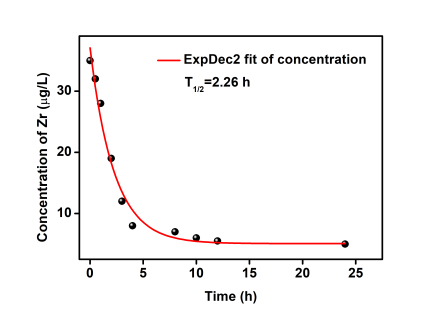
**

**Figure S31.** Blood circulation lifetime of **ZTCH** after intravenous injection.

**
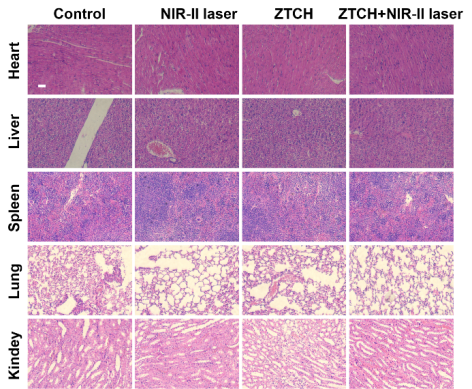
**

**Figure S32.** The H&E staining histological sections of major organs of ICR mice bearing H22 tumor of different groups after varied treatments (PBS, NIR laser, **ZTCH** with/without NIR laser irradiation). Scale bar: 500 μm.


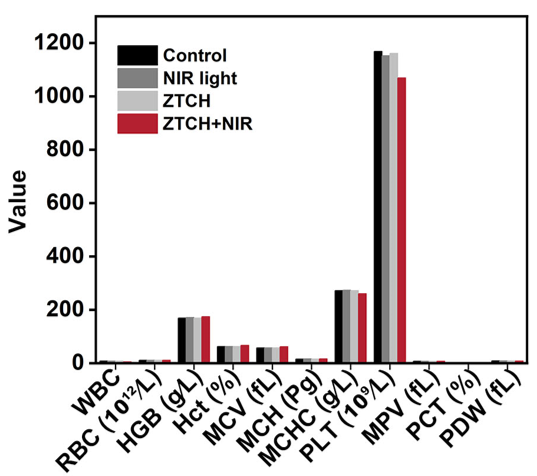


**Figure S33.** The hematology data which were achieved from mice in different groups.


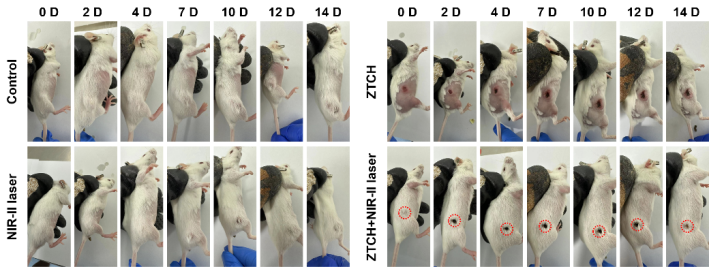


**Figure S34.** Photos of mice from control, NIR-II laser irradiation, **ZTCH** and **ZTCH** with NIR-II laser irradiation groups, wherein tumor growths were compared at different days during the treatment.


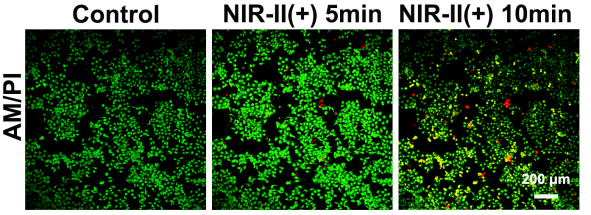


**Figure S35.** CLSM images of HepG2 cells treated with **ZrTc@HA** (50 μg/mL) under different irradiation time, stained with calcein AM/PI (λ_ex_ = 1250 nm, laser power: 0.1 W/cm^2^).


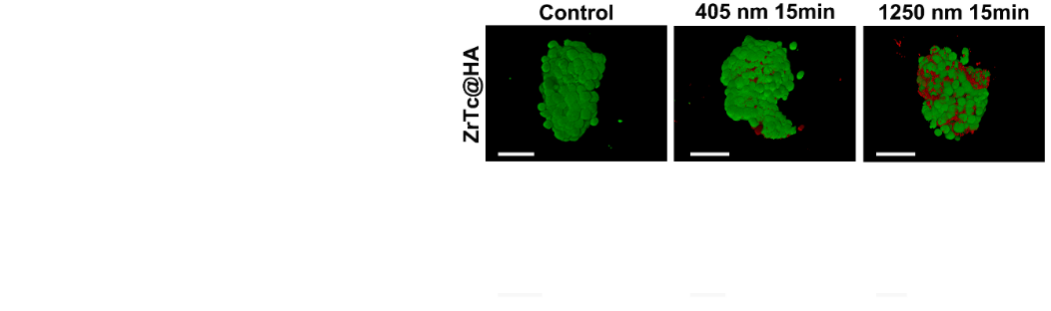


**Figure S36.** CLSM images of HepG2 MCSs treated with **ZrTc@HA** (50 μg/mL) and incubated with calcein AM/PI. HepG2 MCSs were irradiated by 405 nm, and 1250 nm laser for 15 min (fixed power: 0.1 W/cm^2^, scale bar: 100 μm).


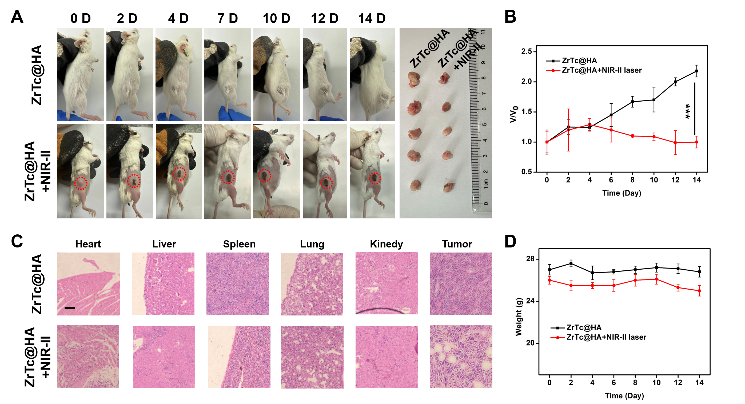


**Figure S37.** (A) Photos of mice and tumors from **ZrTc@HA** without/with NIR-II laser irradiation groups. (B) Spectrum of tumor volumes of **ZrTc@HA** without/with NIR-II laser irradiation groups (n = 5 independent experiments). (C) The H&E staining histological sections of major organs of ICR mice of different groups after varied treatments (**ZrTc@HA** without/with NIR-II laser irradiation). Scale bar: 500 μm. (D) Spectrum of body weights of female ICR mice of **ZrTc@HA** without/with NIR-II laser groups (n = 5 independent experiments). Statistical analysis was conducted with unpaired t-test analysis (ns, not significant, *P < 0.05, **P < 0.01, ***P < 0.001).

**Table S1.** Activity comparison between **ZTCH** and other reported NIR photocatalysts.

|  | **ZTCH** | **RPCN** | **CNCPF** | **SnS_1.68_-WO_2.41_** |
| --- | --- | --- | --- | --- |
| Multiphoton photocatalyst | yes | not | not | not |
| Band gap (eV) | 2.34 | 1.71 | 1.04 | 1.49 |
| λ_ex_ (nm) | 1250 | 808 | 808 | 808 |
| H_2_ production activity | 21.54 μM (7.3 μL) | 70 ng/g | 10 μL | 0.6 μg/g |
| Irradiation time | 4 min (50 μg/mL) | 20 min (200 μg/mL) | 30 min (200 μg/mL) | 20 min (100 μg/mL) |
| Fluorescence imaging | Yes | No | No | No |
| Ref. | This work | Adv. Mater. 2021, 33, 2101455 | Adv. Mater. 2024, 36, 2308774 | Nat. Commun., 2021, 12, 1345 |

**Table S2.** Tumor volumes of the control, NIR-II laser, and **ZTCH** with/without NIR-II laser groups during 14 days.

|  | 0 Day (mm^3^) | 2 Day (mm^3^) | 4 Day (mm^3^) | 6 Day (mm^3^) | 8 Day (mm^3^) | 10 Day (mm^3^) | 12 Day (mm^3^) | 14 Day (mm^3^) |
| --- | --- | --- | --- | --- | --- | --- | --- | --- |
| Control | 125 | 132 | 211 | 281 | 313 | 320 | 325 | 326 |
| NIR laser | 83 | 95 | 110 | 125 | 150 | 192 | 203 | 208 |
| **ZTCH** | 60 | 69 | 85 | 120 | 160 | 162 | 180 | 189 |
| **ZTCH**+NIR laser | 73 | 79 | 70 | 37 | 20 | 19 | 17 | 11 |

**Table S3.** Tumor volumes of **ZrTc@HA** and **ZrTc@HA** with NIR-II laser groups during 14 days.

|  | 0 Day (mm^3^) | 2 Day (mm^3^) | 4 Day (mm^3^) | 6 Day (mm^3^) | 8 Day (mm^3^) | 10 Day (mm^3^) | 12 Day (mm^3^) | 14 Day (mm^3^) |
| --- | --- | --- | --- | --- | --- | --- | --- | --- |
| **ZrTc@HA** | 145 | 181 | 180 | 210 | 242 | 247 | 290 | 320 |
| **ZrTc@HA**+NIR laser | 177 | 212 | 228 | 212 | 195 | 193 | 175 | 170 |

**References**

[1] B. Li, X. Lu, Y. P. Tian, D. D. Li, *Angew. Chem. Int. Ed.* **2022**, *61*, e202206755.

[2] a) X. B. Li, Y. Hu, F. Dong, J. T. Huang, L. Han, F, Deng, Y. D. Luo, Y. Xie, C. Z. He, Z. J. Feng, Z. Chen, Y. F. Zhu, *Appl. Catal. B-Environ.* **2023**, *325*, 122341; b) Y.-H. Liu, X. Y. Chu, Y. X. Jiang, W. Han, Y. Wang, L.-H. Shao, G. L. Zhang, F.-M. Zhang, *Adv. Funct. Mater*. **2024**, *34*, 2316546; c) S. Q. Zhang, L. L. Lu, J. L. Jiang, N. Liu, B. Zhao, M. M. Xu, P. Cheng, W. Shi, *Adv. Mater.* **2024**, *36*, 2403464.

[3] a) Y. S. Xu, M. J. Fan, W. J. Yang, Y. H. Xiao, L. T. Zeng, X. Wu, Q. H. Xu, C. L. Su, Q. J. He, *Adv. Mater.* **2021**, *33*, 2101455; b) C. Z. Yang, J. S. Zhang, M. Y. Chang, J. Tan, M. Yuan, Y. L. Bian, B. Liu, Z. D. Liu, M. F. Wang, B. B. Ding, P. A. Ma, J. Lin, *Adv. Mater.* **2024**, *36*, 2308774; c) B. Zhao, Y. S. Wang, X. X. Yao, D. Y. Chen, M. J. Fan, Z. K. Jin, Q. J. He, *Nat. Commun.* **2021**, *12*, 1345.
